# Selected prebiotics and synbiotics administered *in ovo* can modify innate immunity in chicken broilers

**DOI:** 10.1186/s12917-019-1850-8

**Published:** 2019-04-03

**Authors:** Tadeusz Stefaniak, Jan P. Madej, Stanisław Graczyk, Maria Siwek, Ewa Łukaszewicz, Artur Kowalczyk, Marcin Sieńczyk, Marek Bednarczyk

**Affiliations:** 1 Department of Immunology, Pathophysiology and Veterinary Preventive Medicine, Faculty of Veterinary Medicine, Wrocław University of Environmental and Life Sciences, Norwida 31, 50-375 Wrocław, Poland; 2 Department of Histology and Embryology, Faculty of Veterinary Medicine, Wrocław University of Environmental and Life Sciences, Norwida 25, 50-375 Wrocław, Poland; 3 Department of Animal Biotechnology and Genetics, UTP University of Science and Technology, Mazowiecka 28, 85-084 Bydgoszcz, Poland; 4 Institute of Animal Breeding, Wrocław University of Environmental and Life Sciences, Chełmońskiego 38C, 51-630 Wrocław, Poland; 5 Division of Medicinal Chemistry & Microbiology, Faculty of Chemistry, Wroclaw University of Technology, Wrocław, Poland

**Keywords:** Prebiotic, Synbiotic, Chicken, *In ovo*, Innate immunity

## Abstract

**Background:**

A previous study showed that prebiotics and synbiotics administered *in ovo* into the egg air cell on the 12th day of incubation enhance the growth and development of chickens. However, the influence of this procedure on the development and efficiency of the innate immune system of broiler chickens is unclear. Therefore, the aim of this study was to evaluate whether the early (on the 12th day of embryo development) *in ovo* administration of selected prebiotics (inulin − Pre1 and Bi^2^tos − Pre2) and synbiotics (inulin + *Lactococcus lactis* subsp. *lactis* IBB SL1 − Syn1 and Bi^2^tos + *L. lactis* subsp. *cremoris* IBB SC1 − Syn2) influences the innate immune system.

**Results:**

Chickens (broiler, Ross 308) that were treated with Pre1 exhibited a decreased H/L ratio on D7, but an increased H/L ratio was observed on D21 and D35. In the remaining experimental groups, an increase in the H/L ratio was observed on D21 and D35. The oxidative potential of leukocytes measured using the NBT test increased on D21 in Pre2 and Syn1 groups. The rate of the phagocytic ability of leukocytes increased in Pre1 and Syn1 groups on D21. The phagocytic index decreased in Pre1 and Syn2 groups on D21 and D35. Concurrently, the count of WBC in circulating blood decreased on D21 in Pre1, Pre2, and Syn1 groups. The hematocrit value was increased in Syn1 chickens on D21, in Pre1 chickens on D35, and in Syn2 chickens on both time points.

**Conclusions:**

Early *in ovo* treatment of chicken embryos with prebiotics and synbiotics may temporarily modulate not only the production/maturation of leukocytes but also their reactivity.

**Electronic supplementary material:**

The online version of this article (10.1186/s12917-019-1850-8) contains supplementary material, which is available to authorized users.

## Background

### Prebiotics

Diets enriched in nondigestible carbohydrates (prebiotics) stimulate gut-associated lymphatic tissue [1] and increase the metabolic activity and development of beneficial bacteria in the colon [2, 3]. Prebiotic inulin administered orally with selected postbiotics in broiler chickens positively influence weight gain, feed efficiency, and mucosal architecture [4]. Diets enriched in prebiotics reduce toxic metabolites and detrimental enzymes such as cycloalkanes, cycloalkenes, and esters (reviewed by Mundi et al. [5]). They also prevent diarrhea and may prevent constipation by the stimulation of intestinal peristalsis and by increasing fecal moisture with osmotic pressure [6]. Prebiotics also alleviate the detoxifying load of the liver, reduce serum cholesterol level and blood pressure, exhibit anticancer activity, and influence the production of vitamins B1, B2, B6, and B12 as well as nicotinic and folic acids [7]. Prebiotics delivered *in ovo* stimulate bifidobacteria proliferation and reduce the number of detrimental microorganisms in the gut [8]. A previous study showed that the prebiotic Bi^2^tos (galacto-oligosaccharide) and synbiotic inulin (fructan) + *Lactococcus lactis* subsp. *lactis* injected *in ovo* into the air cell on the 12th day of embryonic development significantly increased the final body weight of broiler chickens [9]. However, little is known about the influence of *in ovo* administered pre- and synbiotics on the innate immune system.

### *In ovo* administration of pre-, pro-, and synbiotics

To ensure the best protection of the newly hatched chicks, external supplementation should be given as early as possible. The *in ovo* technology enables administration of a given substance in a solution directly into the incubating eggs [6, 10–13]. In our earlier studies, we found that day 12th of incubation is the optimal time for prebiotic injection into the air cell of the incubating egg [13]. This route of prebiotic delivery into an egg air cell ensures that the prebiotic reaches its final destination, i.e., the embryonic gastrointestinal tract [6]. The beneficial effects of early *in ovo* supplementation with bioactive substances (on the 12th day of chicken embryo development—E12) were previously described [8, 10, 14–16]. *In ovo* injection of the prebiotic α-galactoside (RFOs) obtained from *Pisum sativum* L. cv. Opal on the 12th day of incubation leads to long-term maintenance of a high level of intestinal bifidobacteria [8]. The same prebiotic administered in the field condition trial proved that *in ovo* injection could replace antibiotic growth promoter as a non-antimicrobial enhancer additive [10]. Calik et al. [11] injected intraamniotically eggs on the 18th day of embryo incubation with the synbiotic containing inulin and *Enterococcus faecium* and further supplemented diet of broiler chickens with the same synbiotic. This procedure enhanced intestinal integrity, increased cecal beneficial bacterial populations, and cecal butyrate concentration.

### Influence on the innate immune system

Presently, there is limited information on the influence of pre-, pro-, and synbiotics delivered *in ovo* on innate immunity in poultry. Probiotic injection into air cell or into the amniotic fluid on the 18th day of embryo development did not protect against *Salmonella* enteritidis challenge in chickens at 2–3 days of life [17, 18]. Sławinska et al. [19] injected two in-house-developed synbiotics composed of RFO and *L. lactis* subsp. *cremoris* IBB SC1 or *L. lactis* subsp. *lactis* IBB SL1 and a commercial synbiotic Duolac into the air cell on the 12th day of incubation and showed significant changes in cytokine gene expression in the spleen and cecal tonsils. Synbiotic composed of inulin and *L. lactis* subsp. *lactis* IBB SL1 caused downregulation of immune-related gene expression in the cecal tonsils and spleen in chickens [20]. The level of downregulation increased with age and was most likely caused by the stabilization of gastrointestinal microbiota.

As mentioned above, the *in ovo* injection of synbiotics may modulate the immune system of the chicken. According to the literature, this process works probably by stimulating microbiome development in the gut and activating the mucosal immune system through the stimulation of gut antigen-presenting cells that provide protection and regulate immune responses [21]. Previous studies that administered prebiotics and synbiotics *in ovo* on the 12th day of embryo development showed direct (immune system organs) or indirect (gene expression) impact of the bioactive substances on the immune system [14, 20]. However, the mechanisms underlying the interaction of prebiotics and synbiotics with host immune system are not known.

### The aim of the study

The present study therefore aimed to evaluate whether the early (on the 12th day of embryo development) *in ovo* administration of select ed. prebiotics (inulin and Bi^2^tos) and synbiotics (inulin + *L. lactis* subsp. *lactis* IBB SL1 and Bi^2^tos + *L. lactis* subsp. *cremoris* IBB SC1) influence the development and efficiency of the innate immune system of broiler chickens.

## Methods

### Selection and dosing of pre- and synbiotics

The synbiotics were selected from the several combinations of pre- and probiotics by in vitro tests, followed by validation with animal model [22, 23] The optimal doses of pre−/probiotic were selected by evaluating the hatchability and the bacteriological status of the hatched chickens. The highest doses that did not reduce the hatchability (compared with a control group) were determined to be 1.76 mg/embryo for inulin and 0.528 mg/ embryo for Bi^2^tos. The bacteria cultures were prepared as follows: fresh overnight cultures of IBB SL1 and IBB SC1 strains in M17 medium supplemented with 1% glucose (GM17) were used. The number of bacteria was estimated at the level of 3 × 10^8^ of living cells. Before injection, the bacterial cultures were diluted in prebiotic solution to obtain a bacterial suspension of 1000 CFU in 20 μL.

### Material

The experiment was carried out at the experimental farm of Wroclaw University of Environmental and Life Sciences (Wroclaw, Poland). Nine hundred hatching eggs (which weighed approximately 60 g each) were obtained from 32-week-old broiler breeder flock (Ross 308). Eggs were incubated in a commercial hatchery (Drobex, Solec Kujawski, Poland) in Petersime incubator (Zulte, Belgium). After hatching chicks were sexed and male broiler chickens (42.0 g average weight) were used for the study (Table 1).

**Table 1:** Number of embryos and chickens used in experiment

Time	Groups and number of embryos/chickens					Action
	Pre1	Pre2	Syn1	Syn2	C	
E12	800					live embryos
E12	160	160	160	160	160	embryos given to experimental groups and treated *in ovo*
D1	145	137	114	145	130	hatched chickens
D7	− 7	−7	−7	− 7	− 7	randomly selected chickens killed and used to tests
D21	−7	− 7	− 7	− 7	− 7	randomly selected chickens killed and used to tests
D35	−7	− 7	− 7	− 7	− 7	randomly selected chickens killed and used to tests

*C* Control, *Pre1* Prebiotic 1, *Pre2* Prebiotic 2, *Syn1* Synbiotic 1, *Syn2* Synbiotic 2

### *In ovo* treatment

On the 12th day of incubation, the eggs were candled and those with developing embryos were used in the experiment. Then the eggs were randomly divided into five experimental groups (160 eggs each): eggs injected with sterile physiological saline (control group – C); eggs injected with solution containing 1.76 mg of inulin (prebiotic group – Pre1) (Sigma-Aldrich) and 0.528 mg of Bi^2^tos (Pre2) (Clasado Ltd.); and eggs injected with 1.76 mg inulin (synbiotic group – Syn1) and 0.528 mg Bi^2^tos (Syn2) enriched with different probiotic bacteria. Syn1 group received 1000 CFU of *L. lactis* subsp. *lactis* IBB SL1 and Syn2 group received 1000 CFU of *L. lactis* subsp. *cremoris* IBB SC1. The synbiotics injected in Syn1 and Syn2 groups comprised 180 μL of the prebiotic solution and 20 μL of bacterial suspension. An aqueous solution/suspension of bioactive substances, at equal volume of 0.2 mL, was injected into the air cell, and the hole in the egg shell was sealed with the use of special automatic system [10].

### Rearing conditions

Chickens were reared on wood shavings litter till 35th day of age (D35) under uniform, controlled environmental conditions, and in accordance with the recommendations for this line (see Additional file 1). Fresh, good-quality water and commercial feeds were available ad libitum: starter feed from D1 to D21, grower feed from D15 to D28 (blended to reach 100% grower by D21 to gradually change the ration), and then finisher feed up to D35. Basic feed components are presented in Table 2.

**Table 2:** Composition and nutritional values of feeds used for chicken broilers^a^

Ingredients	Starter	Grower	Finisher
Soybean meal	330.3	286.0	256.0
Maize	300.0	300.0	300.0
Wheat grain	262.5	289.1	306.5
Rapeseed meal	30.0	36.0	42.0
Soybean oil	21.1	13.0	18.0
Plant oils	17.0	41.0	49.0
Monocalcium phosphate	14.0	11.7	8.3
Limestone	13.0	11.6	10.5
L-lysine 98	3.1	3.0	2.5
DL-methionine 99	3.0	2.3	1.8
Salt	2.6	2.6	2.5
L-throning	0.6	0.9	0.5
Choline chloride 75%	0.4	0.4	0.4
Vitamin-mineral premix^b^	2.4	2.4	2.4
Calculated nutrient level^b^			
Crude protein (g/kg)	220.0	205.0	195.0
Crude fat (g/kg)	60.6	76.0	88.7
Crude fibre (g/kg)	26.8	26.6	26.7
Ash (g/kg)	61.1	55.8	50.1
Metabolisable energy (kcal/kg)	2980	3100	3200

^a^Feeds were equalized and standardized according to recommendations for chicken broilers [NCR. Nutrients requirement of poultry. 9th rev. ed. National Academy Press, Washington DC, 1994]. ^b^Estimation based on the Polish feedstuff analysis tables [40]

### Blood sampling, hematological analysis, and serum preparation

For hematological analysis and leukocyte functional evaluation on D7, D21, and D35, blood samples were taken from brachial vein of seven chickens from each of the five experimental groups (Table 1). Total white blood cell counts (WBC) were determined in the Bürker chamber, using Natt-Herrick’s solution. Hematocrit (Ht) was measured in heparinized capillary tubes after centrifugation. Blood smears stained according to May–Grünwald–Giemsa were used for leukocyte differential counts. Evaluation was performed using optical microscope. The percentages of particular leukocyte forms were determined by counting 200 subsequently encountered cells and differentiating into lymphocytes, heterophils, eosinophils, basophils, and monocytes. Based on the above, the heterophils–lymphocytes ratio (H/L) was estimated.

To obtain serum, from seven chickens of each group on D7, D21 and D35 the blood was sampled from cervical vein immediately after killing. The serum was separated by centrifugation at 2000×g for 8 min and stored at − 20 °C until analysis. The total serum protein concentration (TSP) was estimated using refractometric method (refractometer Atago, Japan).

The features and potential of the innate immune system were tested using WBC count, H/L ratio, phagocytic ability (rate of phagocytosing leukocytes and phagocytic index), and oxidative potential (reduction of tetrazolium salt).

After experiment the birds were euthanized by decapitation.

### Nitroblue tetrazolium assay

The ability of leukocytes to produce superoxides was evaluated indirectly on D7, D21, and D35, using NBT test (according to Czernomysy-Furowicz and Furowicz [24]). A total of 100 μL of heparinized blood was mixed with 100 μL of 1% nitro blue tetrazolium (NBT, Sigma) in PBS. The samples were incubated for 30 min at 37 °C and subsequently for 30 min in room temperature in humid chamber. After incubation, 50 μL of each sample content was taken in test tubes containing 1 mL of DMSO, and after 10 min of centrifugation at 650×g, the absorbance of supernatant was read at 560 nm (Epoll-20, Poll LTD, Poland). The result was presented as corrected absorbance (CA) calculated for 1000 WBC according to the formula CA = sample absorbance/WBC × 1000.

### Phagocytosis assay

Leukocyte phagocytic ability was performed on the whole heparinized blood taken from 21-day-old- and 35-day-old chickens according to Slapnickova and Berger modified by Pliszczak-Król et al. [25]. One milliliter of blood was mixed with 100 μL of suspension of heat-inactivated *Saccharomyces cerevisiae* cells (8 in McFarland’s scale), and incubated for 20 min at 41 °C. Immediately after incubation two smears were made, dried, and stained according to May–Grünwald–Giemsa. In every smear, 200 leukocytes were evaluated and differentiated as positive (Phag+) or negative (Phag–). Phag+ were defined as cells if at least one yeast cell was seen in the cytoplasm. The phagocytic ability of leukocytes was calculated based on the percentage of Phag+ cells. Additionally, the mean phagocytic index was calculated as the number of ingested yeast cells per 100 leukocytes.

### Statistical analysis

The data were subjected to statistical analysis using Statistica 12.5 software (StatSoft Inc. Tulsa, OK, USA). Significance of differences between the results obtained was appraised using Tukey or Kruskal–Wallis ANOVA test (according to normality of data distribution). A value of *P* < 0.05 was considered significant.

## Results

### Blood hematology

The values of Ht, TSP and WBC were presented in Table 3. On D7 WBC count was the highest in Pre2 group and it differed significantly from Pre1 group (Table 3). On D21, WBC count was almost two times higher in control compared to Pre1, Pre2, and Syn1 groups (*P* < 0.05), but on D35 it did not differ between groups. On D21 Ht value was the lowest in control group and differed significantly from Syn1 and Syn2 groups (*P* < 0.05); the significant difference was also found between Pre2 and Syn1 groups (*P* < 0.05). On D35, Ht value was the lowest in the control group but differed significantly from Pre1, Syn2 groups (*P* < 0.05), and Syn1 group (*P* < 0.001). The values of TSP did not differ on D7 and D21 between groups. On D35 the highest TSP concentration was found in Pre1 group and differed from C and Syn2 groups (*P* < 0.05).

**Table 3:** Mean (±SD) hematocrit (Ht), total serum protein (TSP), and leukocyte count (WBC) in chickens

Groups	Day 7			Day 21			Day 35		
	Ht [%]	TSP [g/L]	WBC [10^3^/μL]	Ht [%]	TSP [g/L]	WBC [10^3^/μL]	Ht [%]	TSP [g/L]	WBC [10^3^/μL]
C	21.6	47.7	32.72	23^a^	51	28.40^a^	27.83^a^	52.3^a^	28.80
	±1.52	±3.8	±4.53	±4.98	±3,6	±5.8	±1.1	±4.4	±8.32
Pre1	21.5	47	32.72^a^	27	54.3	13.33^b^	32.67^b^	62.3^b^	36.40
	±2.07	±3.5	±4.53	±2.0	±8.1	±4.84	±2.73	±3.9	±9.94
Pre2	21	47	39.15^b^	24.33^ac^	53	14.67^b^	31	58.8	54.00
	±1.26	±3.7	±8.27	±2.07	±3.3	±4.13	±1.26	±2.4	±8.72
Syn1	20.33	47.5	30.40	29.67^b^	56.5	14.67^b^	30	51.8^a^	52.00
	±4.72	±1.5	±6.38	±0.52	±2.7	±3.93	±2.1	±4.2	±15.75
Syn2	20.33	44.8	23.59	28.5^bc^	52.8	17.60	31.33^b^	55.3^a^	44.00
	±0.52	±1.9	±3.20	±2.35	±2.8	±2.97	±2.16	±4.1	±7.30

*C* Control, *Pre1* Prebiotic 1, *Pre2* Prebiotic 2, *Syn1* Synbiotic 1, *Syn2* Synbiotic 2. Values within a column with no common lowercase superscripts differ significantly (*P* < 0.05)

### H/L ratio

**Fig. 1:**
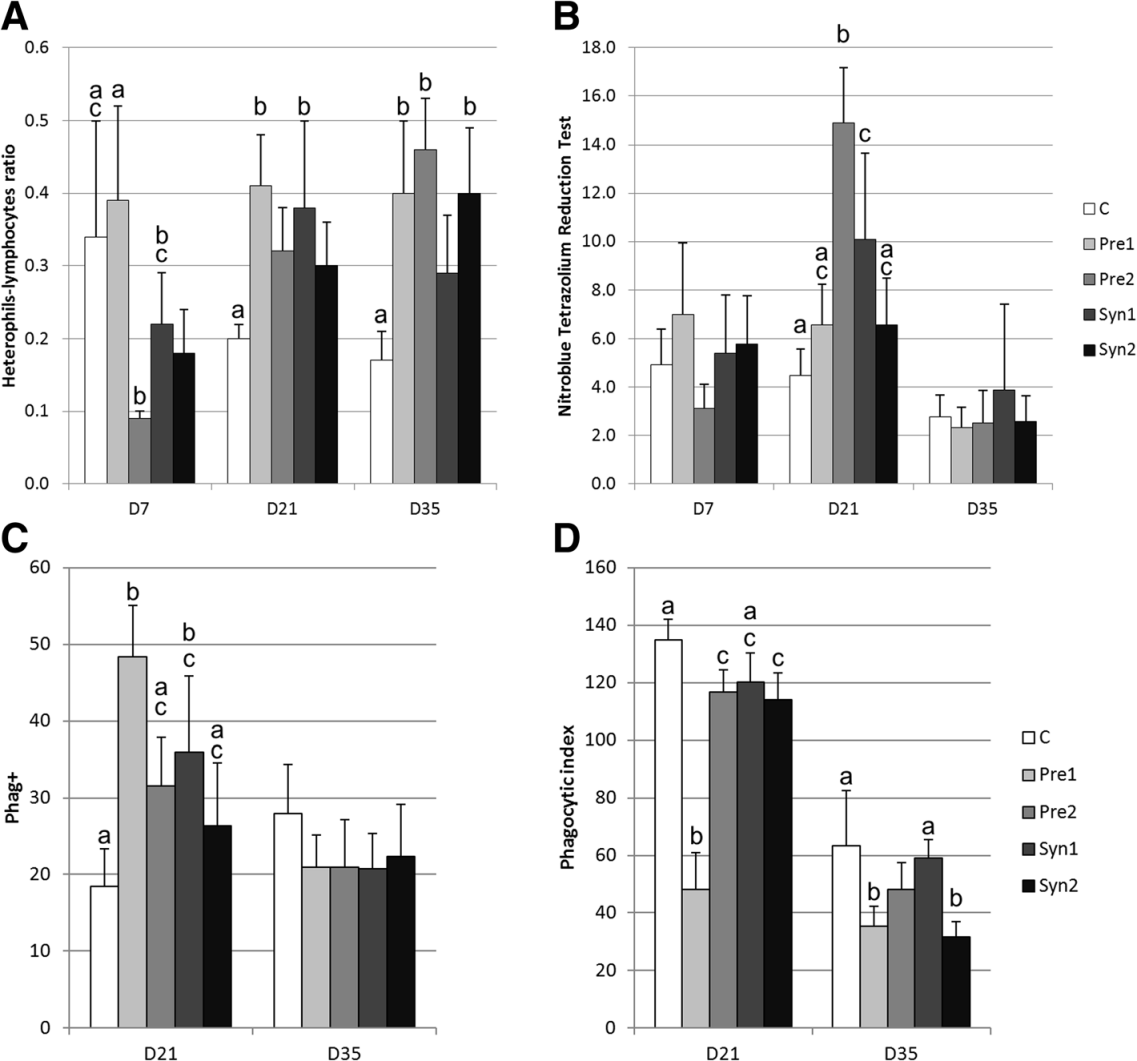
Heterophils–lymphocytes ratio (**a**), nitro blue tetrazolium reduction test (OD = E/1000 WBC × 1000) (**b**), rate of Phag+ leukocytes (**c**), and phagocytic index (number of ingested yeast cells/100 leukocytes) (**d**) in chickens treated *in ovo* with prebiotics and synbiotics (mean ± SD): C – control, Pre1 – prebiotic 1, Pre2 – prebiotic 2, Syn1 – synbiotic 1, Syn2 – synbiotic 2; values within each day with no common lowercase superscripts differ significantly (*P* < 0.05)

In control chickens, the H/L ratio decreased with age (measured on D7, D21, and D35), whereas at the same time points, this ratio increased linearly in chickens from Pre2 and Syn2 groups (Fig. 1a), due to the increased concentration of heterophile cells. The H/L ratio was significantly higher in Pre1 and Syn1 groups than in C group on D21 and in Pre1, Pre2, and Syn2 groups on D35 (*P* < 0.05).

### NBT test

In C and Pre1 groups, between D7 and D35, the NBT test-values gradually decreased according to age (Fig. 1b). However, in Pre2 and Syn1 groups, on D21 a significant increase in NBT test-values was observed (*P* < 0.05), but on D35, it returned to the level observed on D7.

### Phagocytosis assay

The rate of Phag+ cells did not differ significantly between D21 and D35 (Fig. 1c); however, in C group it tended to rise, whereas in all prebiotic- and synbiotic-treated groups, it slightly decreased. On D21, the rate of Phag+ cells was found to be the lowest in control group, and it was significantly lower compared to Pre1 (*P* < 0.001) and Syn1 (*P* < 0.05) groups. At the same time in Pre1 group, the rate of Phag+ cells was significantly higher (*P* < 0.01) than that in C, Pre2, and Syn2 groups but not than in Syn1 group. However on D35 it not differed between groups. The phagocytic index decreased significantly between D21 and D35 in all groups studied (*P* < 0.001) (Fig. 1d). On D21 it was significantly lower in Pre1 than in C group (*P* < 0.001) and in Syn1 (*P* < 0.05) group. On D35 the phagocytic index was significantly lower in Pre1 (*P* < 0.01), Pre2 (*P* < 0.05), and Syn2 (*P* < 0.01) groups than in control. On D21 in inulin-treated chickens, (Pre1) the rate of Phag+ cells was found to be the highest but the phagocytic index the lowest.

Summing up the results obtained we observed that Pre1 and Syn1 treatment results in increase of the values in H/L ratio, NBT test and the rate of Phag+ cells on D21.

## Discussion

The immune system of chickens matures in the course of their embryonic development. Normal development of the immune system occurs under the control of cytokines released by cells of innate and acquired immune systems. It is assumed that chicken’s development occurs in isolation from the external environment. Granulocytes were found in hematopoietic organs as early as on the 12th day of incubation [26]. The appearance of foreign antigens leads to their recognition by pattern recognition receptors (PRRs) and induces an innate immune response. Hence, we suggest that the early (on the 12th day of embryo development) inoculation of pre- and synbiotics into the air cell and their contact with the immune system of the embryo may modify its development.

Our study revealed that on the 21st day, the WBC count was significantly lower in Pre1, Pre2, and Syn1 groups than in control chickens, but the H/L ratio was found to be increased in Pre1 and Syn1 groups and in Pre1, Pre2, and Syn2 groups on D35 (Fig. 1a). In contrast, laying hens fed with prebiotic IMO (≥45% of isomaltose, isomaltotriose, and panose), probiotic PrimaLac® (lyophilized mixture containing 1 × 10^9^ cfu/g of *Lactobacillus acidophilus, Lactobacillus casei, Bifidobacterium bifidum, Streptococcus faecium,* and *Aspergillus oryzae*), or with synbiotic (both above pre- and probiotic mixed) had reduced levels of heterophil percentage and H/L ratio and increased lymphocyte percentage at 36 and 52 weeks of age [27]. Zulkifli and Siegel [26] found that H/L ratios decreased very rapidly after hatching, from 1.76 at the day of hatching to 0.39 on D8. In older chickens, the number of blood lymphocytes decreased and the number of heterophils increased in response to stressors followed by increased levels of corticosterone in the chicken [28, 29]; therefore, the H/L ratio was evaluated as a good stress indicator. In our study, the chickens of all groups were maintained in similar and comfortable conditions; therefore, the increase in the H/L ratio might not be due to stress but rather due to the changes in leukocyte maturation [30, 31] or enhanced colonization of secondary lymphatic organs by lymphocytes [15] caused by early, *in ovo* treatment. These findings, however, disagree with the results of Kim et al. [32] who indicated that prebiotics (mannan-oligosaccharides and low doses of fructooligosaccharides) delivered in food decrease the H/L ratio in broiler chicks. This difference may result from time and route of administration of prebiotics.

Innate immunity mechanisms of the chicken embryo are able to respond to foreign antigens delivered *in ovo* at early stages of development. For example, Petrone et al. [30] observed stimulated granulopoiesis in the chicken yolk sac membrane after inoculation of herpesvirus (HVT) vaccine into the yolk sac on the 10th day of embryo development. The authors suggest that the administration of an antigen such as a virus at an early stage of embryo development can stimulate granulopoiesis in the yolk sac, and influence also the emergence of granulocytes in embryonic liver and chorioallantoic membrane.

Cetin et al. [31] observed that the probiotic-supplemented feed caused a significant increase (*P* < 0.05) in the hematocrit value in turkey, but mannan-oligosaccharide (MOS) supplementation did not change this parameter. In our study, Pre1 and Syn2 groups showed higher Ht values than the control group on D35 (Table 3). This finding shows that *in ovo* administration of prebiotics and synbiotics may improve *hematopoiesis.*

The results of this study showed that the ability of leukocytes to produce oxygen-free radicals decreased according to the age of chickens. However, the concurrent increase in superoxide production and phagocytic ability enhanced only on D21 in the Syn1 group. It is tempting to suggest that in the group that received inulin + *L. lactis* subsp. *lactis* IBB SL1, the ability to ingest and kill the potentially pathogenic microorganisms also increased. A partial effect was achieved on D21 in the Pre2 (Bi^2^tos-treated) group where the NBT reduction ability increased and in the Pre1 (inulin-treated) group where the phagocytic ratio increased significantly. Surprisingly, in the Syn2 (Bi^2^tos + *L. lactis* subsp. *cremoris* IBB SC1-treated) group, both parameters did not differ with those in the control group. On the basis of these results, we suggest that both the studied prebiotics and Syn1 may temporarily modulate not only the production/maturation of leukocytes but also their reactivity.

Farnell et al. [33] showed the escalation of antioxidative potential and enhanced degranulation of chicken heterophils 24 h after probiotic treatment. Similarly, Olivares et al. [34] found that in humans who were fed with lactic acid bacteria or with commercial yogurt, the number of circulating phagocytes and their activity increased. Parra et al. [35] detected that in humans who were fed with fermented milk, a triggered oxidative burst occurred in monocytes, and concurrently, the activity of natural killer cells increased. Higgins et al. [36] determined the influence of probiotics administered within one hour after the experimental challenge of chickens challenged on the day of the hatch with *Salmonella* Enteritidis on the count of intestinal macrophages and the phagocytosis of this bacterial serovar by peritoneal exudate cells in vitro. They showed that although significant differences occurred in the number of macrophages, the bacterial count was reduced in cecal tonsils within 24 h. Bandyopadhyay and Das Mohapatra [37] reported the impact of probiotic supplementation on phagocytic ratio and phagocytic index in fish. These authors performed an experiment with *Bacillus circulans* used as a probiotic supplement in the feeds for the fingerlings of *Catla catla*. Their experiment showed that the administration of 2 × 10^5^ *B. circulans* cells per 100 g feed for 60 days at 5% of the body weight per day significantly increased the phagocytic ratio and phagocytic index.

The results discussed here revealed that *in ovo* inoculation of prebiotics and synbiotics caused a transient increase in the rate of phagocytosing cells in 21-day-old chickens, although the number of ingested yeast cells did not differ significantly from that in the control group. On D35, no such difference was observed, and the Phag+ cell ratio was found to be similar in both control and experimental groups (Fig. 1c, d).

Many reports emphasize that the administration of inulin (IN) exerts positive effects on immune response and health condition. As reviewed by Kozlowska et al. [38], inulin supplementation in monogastric animals may have indirect and direct effects. Indirect impact refers to the stimulation of the development of healthy intestinal microbial strains, which in turn inhibit the proliferation of pathogenic bacteria that may cause infections and produce toxins harmful to the organism. Direct effect influences the activity of phagocytic cells as well as nonspecific mechanisms of humoral immunity.

In addition to the synbiotics presented in this study, our previous experiment, based on other bioactive substances, also proved their immunomodulatory effects [16, 19]. A significant upregulation of gene expression of IL-4, IL-6, IFNβ, and IL-18 and downregulation of IL-12 gene expression were observed in spleens of chickens treated with *L. lactis* subsp. *cremoris* IBB SC1 with RFO compared to control [19].

To the best of the author’s knowledge, the present study is the first to describe the impact of prebiotics and synbiotics administered *in ovo* on the functioning of the innate immunity in embryos and chickens in the first weeks of life. The available data have reported only selected indicators of immunity, which made the discussion of our results difficult. Moreover, the effect of prebiotics or synbiotics in animals depends on many factors such as sources of microbiota, doses, the frequency of administration, chemical contaminations, environmental conditions (elimination of stressors), and the route and method of administration [39].

In chickens treated *in ovo* with Pre1 (inulin), a significant increase in the number of Phag+ cells and a decrease in the phagocytic index were observed on the 21st day of life. Cellular oxidative potential measured by NBT reduction was significantly higher in Pre2- and Syn1-treated chickens on D21.

## Conclusions

Both the studied prebiotics and Syn1 inoculated *in ovo* on the 12th day of embryo development may temporarily modulate not only the production/maturation of leukocytes but also their reactivity. Pre1 and both synbiotics induced a significant increase in Ht in chickens on D21 and/or D35. These findings indicate the stimulatory effect of the tested prebiotics and synbiotics on hematopoiesis. Further studies are necessary to explain the mechanisms of the observed influence of the prebiotics and synbiotics on the innate immune system.

## Additional file

Additional file 1: **Table S1.** Environmental conditions used for chicken broilers Ross 308 (DOCX 19 kb)

## Abbreviations

D7/D21/D35: day 7/21/35 after hatching
E12: 12th day of incubation
H/L: Heterophils–lymphocytes ratio
Ht: Hematocrit
NBT: Nitro blue tetrazolium assay
TSP: Total serum protein concentration
WBC: Total white blood cell counts
