# Supplementary material for: Selected prebiotics and synbiotics administered in ovo can modify innate immunity in chicken broilers
Source: BMC Vet Res. 2019 Apr 3;15:105. doi: 10.1186/s12917-019-1850-8 (PMC6448256; doi:10.1186/s12917-019-1850-8)
Supplement: Supplementary file 1 — Table S1. Environmental conditions used for chicken broilers Ross 308 (DOCX 19 kb) [file 12917_2019_1850_MOESM1_ESM.docx]

**Additional file 1. Table 1S.** Environmental conditions used for chicken broilers Ross 308

| Age (days) | Temperature (ºC) | Relative humidity (%) | Lighting program in a 24 hour period  (light/dark)* | Light intensity  (lux) |
| --- | --- | --- | --- | --- |
| 1 | 31 | 60-65 | 24/0 | 40 |
| 3 | 29 |  | 23/1 |  |
| 6 | 28 |  | 22/2 |  |
| 9 | 27 |  | 18/6 | 20 |
| 12 | 26 |  |  |  |
| 15 | 25 |  |  |  |
| 18 | 24 |  |  |  |
| 21 | 23 |  |  |  |
| 24 | 22 |  |  |  |
| 27 | 21 |  |  |  |
| 35 | 20-19 |  |  |  |

*Changes in the length of the lighting program were introduced for 2-3 days.
